# A New Social Picture Task to Assess Interpretation Bias related to social fears in adolescents

**DOI:** 10.1007/s10802-022-00915-3

**Published:** 2022-04-18

**Authors:** Lisan A. Henricks, Wolf-Gero Lange, Maartje Luijten, Eni S. Becker

**Affiliations:** Behavioural Science Institute, Radboud University, P.O. Box 9104, 6500 HE Nijmegen, The Netherlands

**Keywords:** Interpretation bias, Social anxiety, Fear of negative evaluation, Adolescents

## Abstract

This pre-registered study focused on developing a new social picture task to assess interpretation bias related to social fears in adolescents. Using such a pictorial task may increase ecological validity and readily trigger emotional processes compared to more traditional verbal tasks that are often used. In the picture task, ambiguous social pictures were presented, followed by a positive and negative interpretation. In this study, we examined how the new task relates to an already existing interpretation bias task and how the new pictorial task relates to social fears in adolescents. The sample consisted of 329 adolescents aged 12 to 18 years. Interpretation bias was assessed with the newly developed pictorial task and with more traditional verbal vignettes. Social fears were measured with self-report questionnaires. The results suggest that the pictorial task was able to assess interpretation bias comparable to the verbal vignettes, suggesting appropriate convergent validity. Interpretation bias assessed with the picture task was linked to higher levels of fear of negative evaluation, the core symptom of social anxiety, but not to social anxiety symptoms in general. The verbal task was linked to both social fears and thus still seems the preferred method to investigate interpretation bias related to social fears in adolescents. However, we do believe that with further improvement of the pictorial task, it could be a useful addition to the research field.

**Supplementary Information:**

The online version contains supplementary material available at 10.1007/s10802-022-00915-3.

Puberty is a period in which adolescents are extremely sensitive for the opinion of others (Somerville, 2013), leading towards an increase in social fears. Social fears are described by the fear of humiliation or embarrassment in social or performance situations and by being afraid to make a bad impression on others or by possible scrutiny (i.e., fear of negative evaluation [FNE]; American Psychiatric Association 2013). FNE is seen as the core symptom of social anxiety (Turner et al., 1992). Social anxiety, however is a broader concept, and also encompasses the experience of physical symptoms in social situations (e.g., trembling or blushing), and more behavioural symptoms such as avoidance and withdrawal from social situations (American Psychiatric Association, 2013). When left untreated, social fears often follow a chronic pattern leading towards Social Anxiety Disorder (SAD). The mean age of onset of SAD is 15 years (Mancini et al., 2005) and it is one of the most common psychological disorders among adolescents (Costello et al., 2004) with prevalence rates of 8.2–8.6% (Burstein et al., 2011; Kessler et al., 2012). SAD has detrimental consequences: school drop-out, troubled relationships with family, friends and co-workers (Stein & Kean, 2000), other fears, depression, and substance use (American Psychiatric Association, 2013). So, it would be helpful to investigate which factors play a contributing role to get a better understanding of the development and maintenance of social fears.

Many theoretical models assume that biased cognitive processing plays an important (if not causal) role in the onset and maintenance of social fears (Beck et al., 2005; Beck & Clark, 1997; Muris & Field, 2008; Ollendick & Hirshfeld-Becker, 2002; Spence & Rapee, 2016). In fact, the relationship between social fears and cognitive biases could probably better be described as bidirectional, with both factors continuously influencing each other (Mathews & MacLeod, 2002; Wong & Rapee, 2016). Cognitive biases are defined as processing of stimuli in a biased way and are thought to be caused by overactive schemas involving social threat. Different types of cognitive biases exist, but interpretation bias seems to be especially important for social fears. Interpretation bias is described by the tendency to negatively interpret ambiguous social situations (Mathews & MacLeod, 2005). Empirical evidence for the link between interpretation bias and social fears in adolescence is consistently found. A meta-analysis of Stuijfzand et al., (2018) included 27 studies on social anxiety and showed medium positive associations between social anxiety and negative interpretation bias in children and adolescents (*d* = 0.72). The associations increased in strength when youth got older, highlighting the importance to focus on early detection of interpretation bias to prevent its potentially causal role in the development of social fears. At the same time, the association between interpretation bias and social fears was stronger when the content of the ambiguous scenarios addressed social fears, making it important to use interpretation bias tasks specifically including socially threatening stimuli.

## Verbal Vignette Tasks and the Benefits of Pictorial Versions

Up until now, many different tasks to measure interpretation bias exist, with verbal vignettes being the most well-established and widely used type of tasks (Stuijfzand et al., 2018). In these vignette tasks, ambiguous social scenarios are described and individuals have to indicate whether they would interpret the scenario in a more benign or negative manner. Different answer options are possible in vignette tasks such as choosing one interpretation of an offered list (e.g., Creswell et al., 2005), rank-ordering different interpretations (e.g., Amin et al., 1998), rating each interpretation on a scale separately (e.g., Cox et al., 2016), or using an open-ended format in which participants have to write down their own interpretation of the ambiguous situation (e.g., Reid et al., 2006). Some studies also used a combination of these different options (e.g., Miers et al., 2008). Regardless of which answer format is used, interpretation bias is consistently detected in individuals with social fears using such verbal vignette tasks (Stuijfzand et al., 2018).

Despite the fact that these vignette tasks are well-established and able to detect interpretation bias in adolescents with social fears, we believe that replacing verbal vignettes by pictures of ambiguous social scenarios could be beneficial as it may increase the ecological validity and social salience of the task. Social situations are often complex and the interpretation of a situation is highly dependent upon the context (Gaskell & Marslen-Wilson, 2001). Pictures may be a more naturalistic representation of these complex interactions and provide more information than a short abstract verbal description for instance via facial expressions, gestures, body postures, or situational cues (Haller et al., 2016).

A more controversial argument for the use of pictorial stimuli is the idea that adding pictures may increase self-relevance and mental imagery. For instance, research showed that adding visual cues might be helpful to enhance imagination and to trigger emotional processes, including interpretation biases (Pictet & Holmes, 2013). This can be explained by the fact that visual stimuli are faster to be processed than verbal stimuli as they have immediate access to affective systems in the brain, while verbal stimuli first have to be processed by another brain system, the so-called lexicon (De Houwer & Hermans, 1994). However, there is actually an ongoing debate about this issue because it could also be that because pictures provide more details, they, in fact, leave less room open for imagination. For instance, participants experienced more difficulties engaging with unfamiliar visual stimuli than with verbal stimuli (Lisk et al., 2018), and they found it harder to vividly imagine pictorial scenes (De Voogd et al., 2017).

Another advantage of this pictorial task compared to the vignette task is that it relies less heavily on literacy skills of adolescents. The description of the ambiguous scenario is replaced by a picture. Interpretations can still be presented in a verbal form, but these sentences are relatively short and easy understandable. A picture task thus asks to a lesser extent the understanding of abstract verbal information. This can especially be helpful for younger adolescents, or for adolescents with learning difficulties such as dyslexia, a common problem as 11.6% of children without a family history of reading problems are diagnosed with dyslexia (Snowling & Melby-Lervåg, 2016).

Pictorial tasks to measure interpretation bias can also be valuable for intervention which aim to modify interpretation bias (i.e., Cognitive Bias Modification – Interpretation; CBM-I). Up until now several attempts have been made in CBM-I, both using pictorial as well as verbal trainings (e.g., De Voogd et al., 2017; Lisk et al., 2018). Interestingly though, the pre-post effectiveness of the trainings was assessed in both studies using verbal interpretation bias tasks only. This is problematic as it required a larger transfer-effect from training to interpretation bias measure for the pictorial version than for the verbal version of the training. Indeed, in the study of (De Voogd et al., 2017), it was found that the verbal CBM-I version was more effective in reducing negative interpretation bias, which could have been a side-effect of the modality of the assessment task which more closely matched the verbal training. By developing a pictorial task to assess interpretation bias, we could thus more validly investigate the effectiveness of pictorial CBM-I trainings.

## Social Picture Tasks

Up until now, three studies developed a social picture task to assess interpretation bias related to social fears in children and adolescents. One of these studies used a combination of verbal and visual descriptions of ambiguous scenarios for children from 5 to 9 years old (Creswell et al., 2011). Specifically, the description of the ambiguous scenarios was spoken and accompanied by a cartoon representation of the situation. Children were then presented with two cartoons explaining the situation, a threatening and a non-threatening interpretation, and had to indicate which interpretation would be most likely. Results of this study showed that children with more threat interpretations experienced more anxiety levels at the same time point. Adding these cartoons facilitates the understanding and imagination of the situations, but does not improve the ecological validity of the task as cartoons are more simple and less naturalistic representations of social situations.

In another study with children and adolescents aged 7 to 13, pictures were used without any verbal descriptions (In-Albon et al., 2008). Socially ambiguous pictures were shown and participants had to indicate as fast as possible whether the picture represented a popular or unpopular child by pressing a button. Their results showed that children with social anxiety had the tendency to categorize children in ambiguous social pictures as unpopular and thus negatively interpreted the situation. The stimuli in this study however did not promote self-relevance: children were not instructed to imagine themselves in the ambiguous situation, but just had to rate the actor in the situation. This may not be optimal since negative interpretations are especially triggered in self-relevant situations (Vassilopoulos & Banerjee, 2012).

Finally, Haller et al., (2016) developed a social picture task for adolescents between 14 and 17 years old. They created pictures of everyday situations and added an image of the back of the participant into each picture to fabricate the participant within the social scene. By doing so, the authors aimed to facilitate mental imagery and enhance self-relevance with the situation. The scenes were primarily situated in and around medieval school buildings with more classical interiors common in the United Kingdom. Three verbal interpretations were presented: a positive, a negative and a neutral one, with the latter interpretation being unrelated to the participant. For each interpretation, participants had to indicate on a Likert scale how likely they were to interpret the situation in this way. Afterwards they were forced to select the interpretation they perceived as most likely. Results showed that adolescents with increased social anxiety were more likely to have negative interpretations and less likely to have positive interpretations than adolescents with lower levels of social anxiety.

In sum, while offering more ecological validity and readily triggering emotional and related interpretational processes, few pictorial versions of interpretation bias measures have been developed to assess cognitive distortions in youth. These existing picture tasks are however not suitable for investigating interpretation bias in a more general Western-European adolescent sample as they either targeted child samples (Creswell et al., 2011; In-Albon et al., 2008) or were specifically tailored to adolescents in the United Kingdom (Haller et al., 2016). To overcome these issues, we developed a social picture task similar task to Haller et al., (2016), with socially ambiguous pictures of daily school scenes for adolescents accompanied by a verbal positive and negative interpretation of the situation. Besides the scenes, there were also some other differences compared to the task of Haller et al., (2016). Specifically, we did not include neutral interpretations unrelated to the participant, because we preferred the interpretations to resolve the ambiguity of the situation. Also, after seeing a picture, first, adolescents had to select the interpretation they found matching the picture scenario best and, second, rated each interpretation in terms of how likely they found the interpretation matching the scenario. We chose this specific order, because we wanted to first measure the more automatic impulsive response and afterwards the more deliberate response to the interpretations. Finally, instead of making a sophisticated personalized version of the task by using pictures of participants themselves, like Haller et al., (2016) did, we used a more simplified method to enhance self-relevance. Specifically, we selected scenes in which the actors were looking towards another person who was not present in the picture itself at all, or only for a small part (e.g., only an arm was visible). In this way, participants could imagine that they were in that person’s position. This resulted in a pragmatic and standardized task which can also be used for online or anonymous studies. In the current study we investigate whether this social picture task is an appropriate method to assess interpretation bias in adolescents.

## Current Study

The first aim of this study was to investigate how our new social picture task to assess interpretation bias is related to a more traditional verbal vignette task of interpretation bias. We expected at least a moderately positive significant correlation between the interpretation bias scores of the social picture task and the verbal vignette task. Individuals with more negative interpretation bias measured with the verbal vignettes were expected to also show more negative interpretation bias on the social pictures. The second aim of the study was to examine whether adolescents with social fears have a more negative interpretation bias on the pictorial and verbal vignette tasks. We hypothesized that individuals with higher fear of negative evaluation (i.e., the core symptom of social anxiety) and/or more general social anxiety symptoms would show more negative interpretation bias scores on both the pictorial and verbal vignettes.

For exploratory reasons we were also interested whether there are sex differences in the association between social fears and interpretation bias measured with both tasks. Previous research showed that girls experience more negative interpretation bias than boys (Gluck et al., 2014) and social fears are more likely to be experienced by girls than boys (Asher et al., 2017). However, it remains unclear whether the link between social fears and interpretation bias is also different for boys and girls. The current study examined this exploratory question without forming specific hypotheses^1^.

## Methods

This study is pre-registered. For more information and all details about the study, see: https://osf.io/b35da/.

## Sample

Participants were recruited in different ways. For instance, we sent an e-mail or text message to school directors/teachers who we know from previous research collaborations. We also came in touch with several other school directors, via some of our friends or family members working in high schools. School directors were asked whether they could put the information about the study on their school web page, or distribute the information to the parents and adolescents via e-mail. In total, four of the seven contacted schools agreed to do this. These four secondary schools were located in different regions of the Netherlands (three in different cities in the Western, Middle and Eastern part and one school in a more rural area).

In total, 412 adolescents in the Netherlands participated in the online study. Participants who did not complete the main variables of interest (i.e., interpretation bias, social fears) were excluded (*n* = 8). Some adolescents completed the measures in less than 15 min. We did not consider their answers as valid and they were therefore excluded from further analyses (*n* = 57) as well. Some of these adolescents participated again in the study and took more than 15 min to complete the measures the second time. Their answers of the second time were taken into account^2^. There were also individuals who completed the measures twice and took both times more than 15 min to complete them, probably because they wanted to receive the monetary reward twice. For those participants, we only considered the data from the first time they participated (*n* = 5). One participant was too old for the study (19 years) and was excluded from the analyses. Finally, we ran some reliability checks to identify individuals who answered in a ‘straight-line’ manner (i.e., they choose the same extreme answer most of the time). We identified these participants by looking at the raw data of two self-report scales (i.e., Brief Fear of Negative Evaluation [BFNE], Rosenberg Self-Esteem Scale [RSES]) which contained reversed items. Participants who rushed through the measures inattentively, may have filled in the same score to all items without noticing the reverse items (e.g., they could have simultaneously confirmed ‘*I am positive about myself*’ and ‘*I feel like a failure*’). We excluded participants who filled in the same extreme score to more than 25% of the reverse-coded items compared to the non-reversed items of the BFNE and/or RSES (*n* = 12).

After excluding these participants, the final sample consisted of 329 adolescents (40.1% boys) between 12 and 18 years (*M*_age_ = 15.08, *SD*_age_ = 1.76). The majority of the sample was in secondary school (7.6% in grade 7, 19.5% in grade 8, 17.0% in grade 9, 19.5% in grade 10, 18.8% in grade 11, and 17.0% in grade 12), but 2 adolescents (0.6%) were in higher education. Educational levels of the participants varied between pre-vocational (in Dutch: vmbo, 14.0%), pre-college (in Dutch: havo, 22.5%), pre-university (in Dutch: vwo, 53.8%) or college level (in Dutch: hbo, 0.6%). Some participants followed a combination of different educational levels (9.1%). In the sample, 99.7% fully completed the measures, but one participant had some missing values on the self-esteem items (5 out of the 10 items are missing for this scale). For this person, missing values were replaced using person mean imputation.

## Measures

### Interpretation Bias Social Picture Task

**Fig. 1:**
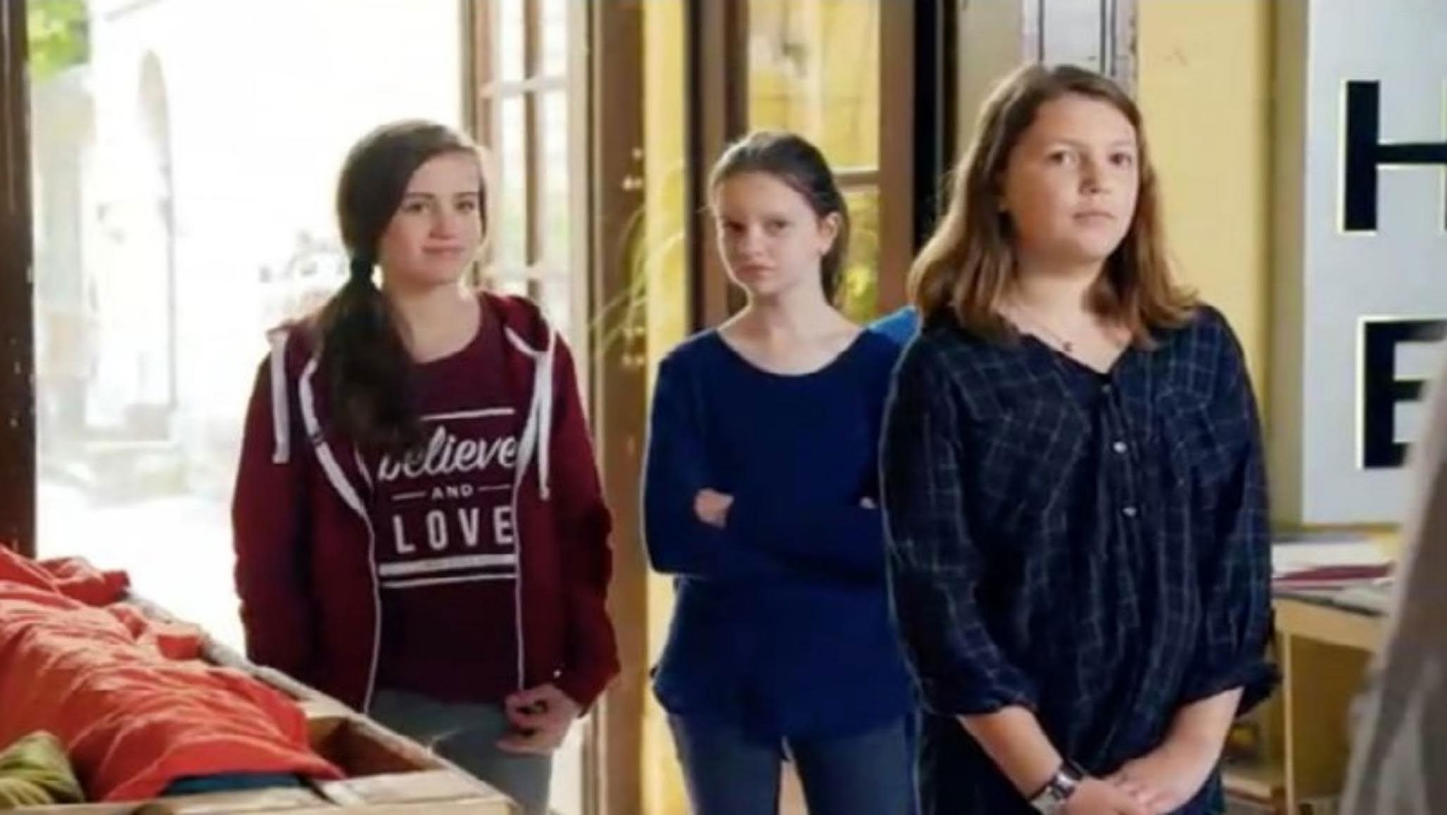
*An Example Trial of the Interpretation bias Social Picture Task, the Schloss Einstein-Radboud Socially Ambiguous Images (SERSAI).* “*New student: You are new at school and they would like to get to know you.*” (positive). “*Strange student: You are new at school, but they don’t want to get to know you*.” (negative)

We developed a social picture task to assess interpretation bias, the Schloss Einstein-Radboud Socially Ambiguous Images (SERSAI). In this task 35 pictures of socially ambiguous scenarios were presented. Each scenario was accompanied by a positive and negative interpretation of the scenario. The pictures were in fact screenshots of ambiguous social scenes selected from a German television show, Schloss Einstein (Saxonia Media, https://www.saxonia-media.de/produktionen/serien/schloss-einstein), regarding students at a high school. Although Germany has a different school system, the setting is quite similar to the Netherlands, making the pictures to be also representative for Dutch adolescents. The two interpretations for each picture were created by the study authors. In order to be able to trigger self-relevance, we used pictures in which the actors looked at another person who was not at all or only partly visible (e.g., only an arm or shoulder) in the picture. This way, participants could be instructed to imagine themselves in that person’s position. We conducted two pilot studies to test the valence of the pictures and the plausibility of the interpretations. For more information on these pilot studies and the selection of stimuli for the SERSAI, please see https://osf.io/b35da/. The materials are available upon request by contacting the second author. Figure 1 presents an example of a trial with a picture of an ambiguous scene and the accompanying positive and negative interpretation. Part A of the Supplementary Materials presents two additional examples.

The interpretation bias social picture task consisted of two different parts. In the first part of the task, adolescents had to indicate which of the two interpretations they found matching the picture scenario best (i.e., the forced-choice part: ‘*Which description do you think best fits the picture?*’). We used block randomization for the forced choice part: each participant was randomly assigned to one of the two equally sized blocks. Both blocks consisted of the same pictures and interpretations. However, for one block the first half of the pictures began with positive interpretations, and the second half of the pictures began with negative interpretations. For the second block, the order of positive or negative interpretations was reversed. Within each block, the different pictures with interpretations were randomly presented to the participant.

In the second part of the task, participants indicated for each interpretation how likely they found the interpretations matching the scenario on a Visual Analogue Scale (VAS) from − 100 = ‘*doesn’t fit at all*’ to 100 = ‘*does fit completely*’ (i.e., the free evaluation part: ‘*How well do you think this description fits the picture?*’). Only one interpretation was presented together with the picture and the VAS to indicate the fit of the interpretation. So, participants saw each picture twice: once with the positive interpretation and once with the negative interpretation. The pictures with interpretations were randomly presented to participants.

The whole social picture task with both the forced-choice and free evaluation parts required 105 responses from participants (i.e., 35 scenarios and three different questions per scenario), which took about 30 min. We calculated two interpretation bias indices. For the forced choice part we computed the percentage of chosen positive interpretations of the total number of situations. Percentages lower than 50% indicated that adolescents had a more negative bias, percentages higher than 50% indicated a more positive bias, and percentages around 50% represented no specific bias for a type of interpretation. For the free evaluation part of the task, we calculated a difference score between the mean score of the responses regarding the fit of the negative interpretations minus the mean score of the responses regarding the fit of the positive interpretations. A more positive score on this index indicated a more positive bias, a more negative score a more negative bias, and a difference score of around zero indicated that an adolescent had no preference for a certain interpretation type. In our sample, the inter-item reliability of the free evaluation part was good (Cronbach’s α = 0.94 for both the positive and negative interpretations).

### Interpretation Bias Verbal Vignette Task

Besides our own pictorial task, interpretation bias was also assessed with the more ‘classical’ approach using verbal vignettes. In total seven vignettes were used from various sources: three were adopted from the Adolescents Interpretation and Belief Questionnaire (Miers et al., 2008), one was from the Interpretation and Judgmental Questionnaire (IJQ; Voncken et al., 2003), one was from an interpretation bias task developed by Mobach et al. (2019), and the remaining two vignettes were created by the study authors. All vignettes described a socially ambiguous scenario and each scenario was accompanied by a positive, negative and neutral interpretation of the scenario. Adolescents rated for each interpretation type how likely they found this interpretation matching the scenario on a 6-point Likert-scale. An example is: ‘*Two classmates talking to each other are looking at you. Why are they looking at you?*’ with the following interpretations: ‘*They say something nice about me*’ (positive), ‘*They are gossiping about me*’ (negative) and ‘*They happen to be looking in my direction*’ (neutral). Part A of the Supplementary Materials presents two additional examples. The vignettes were randomly presented to the participants. An index for interpretation bias was received by calculating a difference score between the mean score of the positive minus the mean score of the negative interpretations. The neutral interpretations were thus not taken into account in this study. A more positive index represented a more positive bias, a more negative score a more negative bias, and a difference score around zero indicated no preference for an interpretation type. In this study, the inter-item reliability was appropriate for positive (Cronbach’s α = 0.76) and negative interpretations (Cronbach’s α = 0.82).

### Social Fears

The Brief Fear of Negative Evaluation Scale (BFNE; Leary 1983) was used to measure fear of negative evaluation, the core symptom of social anxiety. The scale has 12 items and participants indicated the extent to which each item describes themselves. An example item is ‘*I worry about what other people will think of me even when I know it doesn’t make any difference*’. Response categories ranged from 1 = ‘*not at all*’ to 5 = ‘*extremely*’. After reverse coding several items, a mean score was calculated with higher scores indicating higher fear of negative evaluation. The questionnaire has good construct, concurrent and discriminant validity and the test-retest reliability is high (Collins et al., 2005). In this study, the inter-item reliability was very good (Cronbach’s α = 0.93).

The Social Phobia and Anxiety Inventory for Children (SPAI-C; Beidel et al., 1995) was administered to assess adolescents’ more general cognitive, behavioural, and physiological aspects of social anxiety. This questionnaire consists of 26 items describing potential anxiety-provoking social situations. An example item is ‘*I feel scared when I have to join a big group of boys and girls (more than 6)*’. Participants indicated for each item how often they feel anxious in these situations or how often they have anxiety-related cognitions, physiological symptoms or show behavioural avoidance in these situations via a 3-point scale (0 = ‘*never or hardly ever*’, 1 = ‘*sometimes*’, 2 = ‘*most of the times or always*’). Twelve items have sub-items for which first a mean score of the sub-items is calculated to represent these items. Afterwards, we calculated a mean score of all 26 items, a higher score indicated more social anxiety. The SPAI-C has good discriminant and external validity (Beidel et al., 2000). Inter-item reliability of this questionnaire was excellent in our study (Cronbach’s α = 0.97).

### Depressive Mood

We used the 8-item version of the Patient Health Questionnaire modified for Adolescents (PHQ-A-8; Johnson et al., 2002) to control for depressive mood as depression is highly comorbid with social fears and interpretation bias is also found to be an etiological factor for depression (Garber & Weersing, 2010). An example item is ‘*Feeling down, depressed or hopeless*’. Participants indicated their degree of depressive mood in the past two weeks on a scale from 0 = ‘*not at all*’ to 3 = ‘*almost every day*’. A mean score was calculated of all items, with higher scores indicating higher levels of depressive mood. Psychometric properties of this scale are good (Richardson et al., 2010), with good inter-item reliability in our sample (Cronbach’s α = 0.88).

### Self-Esteem

The Rosenberg Self Esteem Scale (RSES; Rosenberg 1965) was used to control for self-esteem as individuals with heightened social fears are featured by low self-esteem (Iancu et al., 2015). This self-report scale contains 10 self-statements which have to be scored on a 4-point scale from 1 = ‘*strongly disagree*’ to 4 = ‘*strongly agree*’. An example item is ‘*On the whole, I am satisfied with myself*’. After reversing the negatively phrased statements, we calculated a mean score of all items. A higher mean score indicated higher self-esteem. The scale has high internal consistency and congruent validity (Franck et al., 2008). In our study, the inter-item reliability of the scale was good (Cronbach’s α = 0.90).

## Procedure

The informed consent and data collection procedure took place online via Qualtrics (https://www.qualtrics.com). For adolescents between 12 and 15 years old, parents first gave active consent, after which adolescents were asked to give online consent as well. After this was done, adolescents could fill in the measures using a separate link. For adolescents between 16 and 18 years old, parental consent was not necessary, we only needed adolescents’ own active consent in order to complete the measures.

Adolescents got a monetary reward once they completed the measures (an online gift card of 10 euros) and took more than 880 s (about 15 min) to finish the study. If they took less time to complete the measures, they received an e-mail request to fill in the measures again. Participants who filled in the measures again received the reward (regardless if they took more or less than 880 s to fill it in). Participants who did not fill in the measures again after the e-mail request did not receive a reward. Participants who filled in the measures twice and took both times more than 880 s, only received a gift card once. The Ethics Committee of the Faculty of Social Sciences, Radboud University in Nijmegen had no formal concerns regarding this research (code: ECSW-2020-083).

### Data Analysis

All analyses were conducted in SPSS version 25. As preliminary step we used Pearson correlation analyses to see how the variables related to each other and we examined the descriptive statistics of the variables. Pearson correlation analyses were also used to test how the different interpretation bias measures were related to each other.

In addition, we conducted a series of hierarchical multiple linear regression analyses to investigate whether adolescents with social fears would show a negative interpretation bias. The different interpretation bias indices were used as dependent variables. Fear of negative evaluation and more general social anxiety symptoms were investigated as main predictors. In all regressions, possible covariates (i.e., depressive mood, self-esteem and age) were entered as predictors in the first block if the Pearson correlations showed that these covariates were significantly and moderately related to the dependent variables of interpretation bias (*p* < .05 and *r* ≥ .30) and if there were no issues of multicollinearity with the main predictors of social fears (VIF < 10, tolerance > 0.10). Beforehand, we checked the assumptions for linear regression, and all assumptions were met for all regression analyses. All tests were two-tailed and we used Bonferroni corrections for multiple testing (i.e., correcting the α-level for significance testing by dividing it by the number of predictors in the regression analyses).

## Results

Descriptive statistics are shown in Tables 1 and 2 presents the Pearson’s correlations.

**Table 1:** Means, Standard Deviations, Minimum and Maximum Scores of all Variables (N = 329)

	*M*	*SD*	*Min*	*Max*
**Interpretation bias social picture task**				
Forced choice (% positive interpretations)	45.86	22.93	0.00	100.00
Free evaluation difference positive – negative	-16.85	62.31	-182.83	157.29
**Interpretation bias verbal vignette task**				
Difference positive – negative	0.09	1.43	-4.29	4.86
**Self-report questionnaires**				
Fear of negative evaluation	35.06	11.56	12.00	60.00
Social anxiety in general	13.61	10.17	0.00	47.33
Depressive mood	15.19	5.36	8.00	31.00
Self-esteem	29.75	5.92	11.00	40.00

**Table 2:** Pearson’s Correlations Between all Variables (N = 329)

	1.	2.	3.	4.	5.	6.	7.	8.
1. Interpretation bias picture task forced choice	--	0.92^***^	0.50^***^	− 0.29^***^	− 0.18^**^	− 0.09	0.24^***^	− 0.19^***^
2. Interpretation bias picture task free evaluation difference		--	0.45^***^	− 0.29^***^	− 0.16^*^	− 0.07	0.24^***^	− 0.19^***^
3. Interpretation bias verbal task difference			--	− 0.50^***^	− 0.48^***^	− 0.33^***^	0.41^***^	0.04
4. Fear of negative evaluation				--	0.65^***^	0.40^***^	− 0.61^***^	− 0.02
5. Social anxiety in general					--	0.43^***^	− 0.55^***^	− 0.20^**^
6. Depressive mood						--	− 0.52^***^	− 0.02
7. Self-esteem							--	− 0.01
8. Age								--
^*^p < .05, ^**^p < .01, ^***^p < .001.								

## Associations Between Different Bias Measures

The Pearson correlation analyses showed that all three different interpretation bias indices of the pictorial and verbal vignette tasks correlated significantly with each other. A more negative bias score on one bias index was related to more negative bias scores on the other two bias indices. Specifically, the forced choice bias score of the social picture task and the interpretation bias score of the verbal vignette task moderately correlated. Similarly, a moderate correlation between the free evaluation interpretation bias score of the social pictures and the interpretation bias score of the verbal vignettes was found. Thus, more negative bias scores on both indices of the social picture task were related to a more negative bias score on the verbal vignette task. Finally, the two interpretation bias indices of the pictorial task highly correlated.

## Negative Interpretation Bias in Adolescents with Heightened Social Fears

We performed three different hierarchical multiple regression analyses to investigate whether individuals with higher social fears (fear of negative evaluation as core symptom of social anxiety and more general social anxiety symptoms) would have more negative interpretation bias measured with the social picture task (separately for the forced-choice and free evaluation part) and verbal vignette task. All regression coefficients of these analyses are shown in Table 3.

**Table 3:** Results of Three Multiple Regression Analyses Predicting Interpretation Bias by Social Fears (N = 329)

	*B*	*B*_*SE*_	β	*t*	*p*
**1. Interpretation bias social picture task forced choice**					
Fear of negative evaluation*	-7.04	1.66	− 0.30	-4.23	< 0.001
Social anxiety in general	0.98	4.10	0.02	0.24	0.812
**2. Interpretation bias social picture task free evaluation difference**					
Fear of negative evaluation*	-21.08	4.51	− 0.33	-4.67	< 0.001
Social anxiety in general	8.92	11.11	0.06	0.80	0.423
**3. Interpretation bias verbal vignette task difference**					
Step 1:					
Depressive mood*	-0.35	0.13	− 0.16	-2.78	0.006
Self-esteem*	0.78	0.14	0.32	5.52	< 0.001
Step 2:					
Depressive mood	-0.18	0.12	− 0.08	-1.52	0.130
Self-esteem	0.18	0.15	0.08	1.18	0.239
Fear of negative evaluation*	-0.41	0.10	− 0.28	-4.15	< 0.001
Social anxiety in general*	-0.81	0.23	− 0.22	-3.45	0.001
*Note.* * Significant predictor in the model.					

### Social Picture Task - Forced Choice

In the first regression, the forced choice index of the pictorial interpretation bias task was the dependent variable and fear of negative evaluation and general social anxiety were entered as predictors. No covariates were taken into account, because the correlations between the dependent variable and the possible covariates self-esteem, depressive mood and age were non-significant and/or small (*p* > .05 and/or *r* < .30). The Bonferroni corrected α-level was 0.025. The model was significant, *F*(2, 326) = 14.45, *p* < .001, *R*^*2*^ = 0.08. Results showed that more fear of negative evaluation significantly predicted more negative bias on this forced choice interpretation bias index. However, more general social anxiety was not a significant predictor of this index.

### Social Picture Task - Free Evaluation

In the second regression, the free evaluation interpretation bias difference score of the pictorial interpretation bias task was the dependent variable. Again, no covariates were taken into account (due to similar reasons as in the first regression), and fear of negative evaluation as well as general social anxiety symptoms were entered as predictors (Bonferroni corrected α-level = 0.025). This model was significant, *F*(2, 326) = 15.27, *p* < .001, *R*^*2*^ = 0.09. More fear of negative evaluation predicted more negative bias, but social anxiety in general did not.

### Verbal Vignette Task

In the third regression, the interpretation bias difference score of the verbal vignette task was the dependent variable. Depressive mood and self-esteem were entered as covariates in the first block as these were significantly and moderately related to the dependent variable (*p* < .05 and *r* > .30). Age was not included as a covariate as it did not relate to interpretation bias measured with the verbal vignettes. Fear of negative evaluation and more general social anxiety were entered as predictors in the second block. The Bonferroni corrected α-level was 0.0125.

The first block with the covariates was significant, *F*(2, 326) = 37.13, *p* < .001, *R*^*2*^ = 0.19. Lower self-esteem and higher depressive mood predicted more negative bias on this scale. After the main predictors were entered in the second block, the model remained significant, *F*(4, 324) = 35.15, *p* < .001, *R*^*2*^ = 0.30. Besides, adding fear of negative evaluation and general social anxiety to the model led to a significant improvement, *F*_change_ (2, 324) = 27.20, *p* < .001, *R*^*2*^_change =_ 0.12. In this block, self-esteem and depressive mood were no longer significant predictors of interpretation bias. Higher levels of fear of negative evaluation and social anxiety in general predicted more negative interpretation bias with the verbal vignette task.

## Sex Differences in the Association Between Social Fears and Interpretation Bias

We also explored whether there were sex differences in how social fears and interpretation bias were related. We created two interaction terms with sex for both fear of negative evaluation and social anxiety symptoms in general. Before creating these interaction terms, the social fears variables were centred. We repeated the three regression analyses described above, but we added the direct effect of sex to the block of the direct effects of fear of negative evaluation and social anxiety in general. Then, we added the two interaction terms in a subsequent block. Bonferroni corrected α-levels were 0.01 for the regressions with the picture task and α = 0.0071 for the vignette task. For all three regressions with the different interpretation bias indices of the pictorial and verbal vignette tasks as dependent variables, neither sex as direct effect, nor the interaction effects of sex were significant. Regression coefficients of these analyses can be found in Table 5 of the Supplementary Materials, part B.

## Discussion

This study investigated whether our newly developed social picture task was an appropriate instrument to assess interpretation bias related to social fears in adolescents. Specifically, we examined how the pictorial task was related to interpretation bias measured with a more traditional verbal vignette task and to levels of fear of negative evaluation (the core fear of social anxiety) and social anxiety symptoms in general. We also explored sex differences in the associations between interpretation bias and social fears.

## Associations Between Different Bias Measures and the Link with Social Fears

Results showed that the new social picture task was moderately correlated with a more traditional verbal vignette task. As expected, both tasks thus measure a similar construct and the pictorial task was able to assess interpretation bias in adolescents. This is a first proof of convergent validity of the pictorial task. Adolescents with higher levels of fear of negative evaluation were found to have a more negative interpretation bias, detected by both the pictorial as well as the verbal task. Contrary to our expectations, the two tasks were differentially related to more general social anxiety symptoms: while adolescents with higher levels of social anxiety in general had more negative interpretation bias on the verbal task, this effect was not found for the pictorial task.

This finding is striking and could imply that the picture task is able to grasp the more cognitive and emotional components of social anxiety, namely fear of negative evaluation, but fails to detect physical and behavioural social anxiety symptoms. This finding makes sense, because the social scenes and the accompanying interpretations used in the task mostly target cognitive components (by asking what adolescents would *think* in these ambiguous situations), but do not ask specifically how adolescents would react or behave in such a situation. This idea was supported by the data as the correlations between the pictorial task and social anxiety in general were mostly non-significant for the behavioural and physical items, but significant for the more cognitive items. However, it can be questioned how valid this explanation is since the verbal task was related to social anxiety in general even though this task also only specifically asks about how adolescents would interpret the situations (so a cognitive component), instead of also targeting behavioural and physical aspects of social anxiety.

A recent meta-analysis investigating interpretation bias in social anxiety revealed larger effect sizes when verbal stimuli were used rather than visual stimuli (Chen et al., 2020). However, the studies using visual stimuli included in this meta-analysis used static pictures or videos of facial expressions, or videos of social situations or behaviours. The type of stimuli in our task (i.e., static pictures of social situations) was thus not taken into account in the meta-analysis. Chen et al., (2020) showed that the effect sizes were specifically smaller for facial stimuli (g = 0.60), while videos of social situations had large effect sizes (g = 0.86) similar to verbal stimuli (g = 0.89–0.99). So their conclusion was mainly driven by the facial stimuli that had been used. Facial stimuli have some disadvantages in measuring interpretation bias as they do not provide a social context, so the stimuli are too simplistic and may not be ecologically valid or do not allow much interpretative space as most facial expressions are accurately recognized (Bijsterbosch et al., 2021; Blanchette & Richards 2010; Chen et al., 2020; Gaskell & Marslen-Wilson, 2001). Therefore, we chose pictures of social scenarios instead. The meta-analysis has also focused on studies with adult participants only. Since it is more difficult for adolescents than adults to imagine themselves in a situation without visual cues (Burnett Heyes et al., 2013), it could be expected that the pictorial task would be able to assess social anxiety in general to a similar degree or produce even larger effect sizes than the verbal task. In sum, we had reason to believe that our pictorial task would be better able to grasp interpretation bias than the verbal tasks. However, this was not supported by our data and our results were in line with the meta-analysis of Chen et al., (2020) after all.

An explanation for the differential links with general social anxiety symptoms between the two interpretation bias tasks could be that the pictorial and verbal task tap into a different type of processing. Pictures are more quickly processed (De Houwer & Hermans, 1994), provide more details and leave less room for own personal imagination than verbal descriptions. This can be compared with reading a book versus seeing a movie: though the story line might be the same, the book leaves more room to own interpretation of the characters and the situation than the movie. Indeed it is found that when reading a text, individuals tend to make a mental image of the information (Denis, 1982) allowing for heightened self-relevance with the verbal vignettes compared to the pictorial task. Specifically, when reading a vignette, individuals may rethink of a situation which occurred to them, filling in the details to the abstract text with personal memories (e.g., thinking of a certain person in your mind when reading the vignettes). As a result of this heightened personal imagination, verbal vignettes perhaps also tap more into memory processes, than merely interpretation bias. Contrary, due to the concrete detailed pictures, there is less room for personal imagination in the pictorial task allowing for a more specific measure of interpretation bias for hypothetical situations with unfamiliar peers. It could therefore be that the pictorial task was only related to the core symptom of social anxiety, fear of negative evaluation, as a response to the specific ambiguous scenario depicted, while the abstract verbal vignettes were also related to more general social anxiety symptoms. Two previous studies investigating the effectiveness of a pictorial version of CBM-I actually support the idea that pictorial stimuli may reduce self-relevance. Specifically, participants had more difficulties engaging with the unfamiliar visual stimuli than with verbal stimuli (Lisk et al., 2018), and that participants in the pictorial training found it harder to concentrate and were not able to imagine the scenes very vividly (De Voogd et al., 2017).

## Sex Differences in the Association Between Social Fears and Interpretation Bias

Contrary to a previous study showing that girls experience more negative interpretation bias than boys (Gluck et al., 2014), we found no sex differences in the levels of interpretation bias. Besides, sex did not play a moderating role in the association between socials fears and interpretation bias in this study. This implies that the cognitive theories regarding interpretation bias and social fears similarly apply to boys and girls, and this idea was also supported by a recent meta-analysis showing that the variance in effect size between interpretation bias and anxiety was not accounted for by sex (Stuijfzand et al., 2018). However, we would like to point out that the sample consisted for a larger part of girls than boys (59.9% versus 40.1%), resulting in an unequal comparison. At the same time, we examined a broad age range of adolescents, from 12 to 18 years old. It could be that sex differences are more pronounced during a specific period in adolescence as a result of pubertal changes (Hayward & Sanborn, 2002). Due to power issues we were not able to investigate sex differences for the specific age groups. Future studies using more balanced sex distributions and examining sex differences for specific age groups should reveal whether the link between interpretation bias and social fears is indeed equal for boys and girls.

## Strengths, Limitations, and Future Directions

The current study was a first step in showing the utility of a social picture task to measure interpretation bias in adolescents. The pictorial task included ecologically valid pictures of daily social interactions between adolescents. Self-relevance was enhanced in this task by using pictures for which participants could imagine the actors were looking at them. One of the major strengths of the study was the large sample of adolescents from varying educational backgrounds.

This study was not without limitations. First of all, due to feasibility issues, we selected the materials based upon pilot studies with adult samples instead of with adolescents. This is not optimal as previous studies showed clear differences between validating a stimuli set based upon child or adult participants (LoBue et al., 2018; LoBue & Thrasher, 2015). Although the final selection of stimuli seems to measure interpretation bias in adolescents, we could re-run the pilot studies with adolescents to examine whether similar stimuli would be selected for the pictorial task. Second, we assumed that adding pictures would be beneficial in order to measure interpretation bias due to enhanced imagination and self-relevance. However, we did not formally test these assumptions, nor did we ask questions about the acceptability and user experience of the task among adolescents. Follow-up studies would benefit by including more subjective questions about these topics in order to draw conclusions about whether or not the pictorial task improved ecological validity and social salience, and whether it is user-friendly. Third, data collection of this study took place during the coronavirus (i.e., COVID-19) pandemic. This study did not include questions regarding the experience of anxiety feelings associated with COVID-19. However, on second thought, this would have been worthwhile due to the fact that COVID-19 had a direct effect on adolescent’s ability to engage in social interactions as high schools were closed and adolescents were not permitted to sport or hang out with their peers. The corona situation could possibly have influenced the results, for instance by increasing levels of social anxiety or depressive mood (Magson et al., 2021). Fourth, it should be noted that the order of the questions in the pictorial task may have impacted the results. Participants first responded to the forced choice part of the pictorial task, before the free evaluation questions were asked. In line with the idea of confirmation bias, participants may have answered the free evaluation part in a more extreme way to match their forced choice responses. Future research should counterbalance the order across participants to investigate this. Finally, all data was cross-sectional, meaning that we cannot draw any conclusions regarding the direction of the relationship between interpretation bias and social fears. By using longitudinal or experimental designs in the future, more straightforward conclusions would be possible.

Future research should formally investigate the psychometric properties of this task by testing for instance its test-retest reliability, content validity and construct validity. The current pictorial task is lengthy (105 responses were needed from participants) as we were interested in how adolescents would interpret different situations and whether the answer format used (forced choice or free evaluation) would matter. We would advise other researchers to shorten the pictorial task for reasons of practicality. This could be done by either using only certain social themes (depending upon the topic of interest), or by using only one answer category as the correlation between the forced choice and free evaluation answers was very high (*r* = .92). If only the forced choice response category is used, it would limit the total responses needed to 35.

Also, it would be beneficial to investigate how different samples react to the social picture task. First, the pictorial task could especially be beneficial for individuals with reading difficulties, such as dyslexia, as it relies less heavily on the understanding of abstract verbal information. It would be interesting to compare how adolescents with limited literacy skills score on the pictorial and verbal vignette task, to see if the picture task is better able to grasp their interpretation bias. Another idea to improve the task for this target group would be to audio-record the interpretations, so adolescents with reading difficulties do not have to read the interpretations themselves. Second, although most studies agree upon the fact that social anxiety can be understood as a severity continuum from shyness to social anxiety disorder (Ruscio, 2010), it would also be important to investigate how the findings of the current study generalize to adolescents with clinical levels of social anxiety.

This picture task may be helpful for researchers who are interested in the link between different cognitive biases, such as attention bias (i.e., the attentional preference for negative stimuli) and interpretation bias. The Cognitive Combined Bias Hypothesis assumes that these biases do not operate in isolation, but rather influence and interact with each other (Hirsch et al., 2006). Up until now most studies failed to investigate this or used different modalities for their attention bias and interpretation bias task (e.g., verbal stimuli for interpretation bias, visual stimuli for attention bias), making it more difficult to investigate the supposed link between cognitive biases. From the current stimuli set, we also selected more positive and negative social situations (instead of only ambiguous situations as used in the current study). These positive and negative stimuli could be used in the future to develop a task to measure attention bias, for instance by using a free viewing paradigm with an eye-tracker while participants select the most fitting interpretation. The current stimuli set thus provides possibilities to measure both attention bias and interpretation bias with the same stimuli, facilitating research on the link between different biases.

## Conclusions

This study contributes to the current field of research by developing a new social picture task to measure interpretation bias. The pictorial task was found to be able to assess interpretation bias in adolescents comparable to the verbal task, hinting at appropriate convergent validity. The social picture task could also identify interpretation bias in adolescents with heightened fear of negative evaluation, the core symptom of social anxiety, but not for adolescents with social anxiety symptoms in general. The traditional verbal task was however linked to both social fears and thus at the moment still seems the preferred method to investigate interpretation bias related to social fears in adolescents.

We assumed that by adding pictures to verbal interpretations of socially ambiguous scenarios, this task increased ecological validity and social salience compared to the more traditional ways of measuring interpretation bias with verbal vignettes. Also, by using pictures to illustrate ambiguous scenarios, this task may have been less dependent upon participants’ literacy skills. However, future research should formally test these assumptions, further validate the new task and test its acceptability among adolescents. Doing so will hopefully help to improve the current pictorial task. If improved in the future, the pictorial task could be beneficial to investigate the link between attention bias and interpretation bias using the same stimuli and it may function as a pre-post measure for pictorial versions of CBM-I. Possibly the task may contribute to a better understanding of the development and maintenance of social fears in adolescence and to eventually be able to prevent and treat mental health problems in later life.

## Electronic Supplementary Material

Below is the link to the electronic supplementary material.

Supplementary Material 1

## Data Availability

All data are available on OSF: https://osf.io/b35da/. We got permission of the producers of the television series Schloss Einstein (Saxonia Media) to share the stimuli material with other researchers upon request.

## Footnotes

1 We also explored whether whether adolescents with social fears experience a negative interpretation bias because they over-interpret ambiguous situations in a negative way, or because they have a lack of interpreting situations in a positive way (Amir et al., 2013; Huppert et al., 2003; Steinman et al., 2020). Results of this exploratory aim can be found in the Supplementary Materials, part C.

2 After reconsideration, we thought it would be possible that certain learning effects could have taken place during this second time. We re-ran the analyses excluding these participants (*n* = 23) to investigate this. However, the results with or without these participants were similar, so we decided to only report the results with these participants included.

## Code availability

Data preparation and analyses have been conducted in SPSS, version 25. All syntax and output files are available on OSF: https://osf.io/b35da/.
