# Correction to: A New Social Picture Task to Assess Interpretation Bias Related to Social Fears in Adolescents

**DOI:** 10.1007/s10802-022-00929-x

**Published:** 2022-05-16

**Authors:** Lisan A. Henricks, Wolf-Gero Lange, Maartje Luijten, Eni S. Becker

**Affiliations:** Behavioural Science Institute, Radboud University, P.O. Box 9104, 6500 HE Nijmegen, The Netherlands

## Correction to: Research on Child and Adolescent Psychopathology https://doi.org/10.1007/s10802-022-00915-3

The original version of this article unfortunately contained some mistakes.

- In the **‘Measures’** section, in the paragraph titled ‘Interpretation bias social picture task’, the website of the German television show Schloss Einstein should be added as a reference. The specific sentence where this website should be mentioned is:
  “The pictures were in fact screenshots of ambiguous social scenes selected form a German television show, Schloss Einstein (Saxonia Media, https://www.saxonia-media.de/produktionen/serien/schloss-einstein), regarding students at a high school.”
- In Fig. 1, there was some text as part of the figure in our original submission. During the publication processes, these sentences were placed at the end of the manuscript (after the conclusion), but these sentences should be placed below Fig. 1:
  “New student: You are new at school and they would like to get to know you.” (positive)
  “Strange student: You are new at school, but they don’t want to get to know you.” (negative)
- In the ‘Acknowledgement’ section, one sentence should be added. Here’s the following corrected statement.

The original article has been corrected.

