# Supplementary material for: A New Social Picture Task to Assess Interpretation Bias related to social fears in adolescents
Source: Res Child Adolesc Psychopathol. 2022 Apr 18;50(8):1067–80. doi: 10.1007/s10802-022-00915-3 (PMC9013980; doi:10.1007/s10802-022-00915-3)
Supplement: Supplementary file 1 — Supplementary Material 1 [file 10802_2022_915_MOESM5_ESM.docx]

**Supplementary Materials**

1. **Example trials of the pictorial and textual interpretation bias tasks**

| **Table 4**  *Two Example Trials of the Pictorial and Textual Task* | | | |
| --- | --- | --- | --- |
|  |  | **Pictorial Task** | **Textual Task** |
| **Example 1** | Scenario | 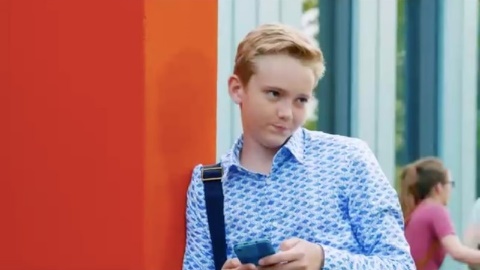 | You are all alone somewhere at a school party and someone you don't know is looking at you. |
|  | Question | Which description do you think best fits the photo? | Why is he/she looking at you? |
|  | Positive  interpretation | Getting attention  He is trying to get your attention so that you will come and talk to him. | He/she likes me and thus tries to get my attention. |
|  | Negative  interpretation | Lonely  He looks at you and thinks you are a loser because you are alone. | He/she notices that I am alone. He/she probably thinks I’m pathetic. |
|  | Neutral interpretation | - | He/she happens to be looking in my direction. |
| **Example 2** | Scenario | 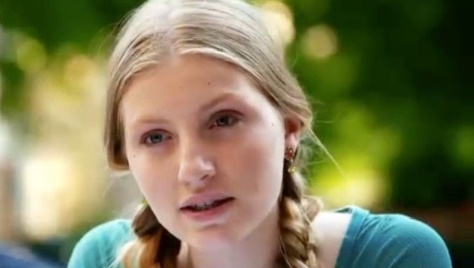 | You are in conversation with someone and you are telling them something. In the middle of a sentence, this person interrupts you. |
|  | Question | Which description do you think best fits the photo? | Why is this person interrupting you? |
|  | Positive interpretation | Interesting  She listens intently to what you have to say. | This person is very interested in what I am saying and wants to know more about it. |
|  | Negative interpretation | Bored  She thinks your story is boring, so her mind is off it. | This person does not find what I am saying fascinating and wants to change the subject. |
|  | Neutral  interpretation | - | This person did not understand something correctly and wants to ask if I can repeat it. |

1. **Exploratory analyses: Sex differences**

We explored whether there were sex differences in how social fears and interpretation bias were related. Table 4 presents the results of the exploratory analyses. All other information regarding this exploratory question is described in the manuscript.

| **Table 5**  *Results of Three Multiple Regression Analyses Examining Sex Differences in the Relation between Social Fears and Interpretation Bias (N = 329)* | | | | | |
| --- | --- | --- | --- | --- | --- |
|  | *B* | *B_SE_* | β | *t* | *p* |
| **1. Interpretation bias social picture task forced choice** | | | | | |
| Step 1: *F*(3, 325) = 10.88, *p* < .001, *R^2^* = .09 | | | | | |
| Fear of negative evaluation* | -7.16 | 1.66 | -.30 | -4.31 | < .001 |
| Social anxiety in general | 0.50 | 4.09 | .01 | 0.12 | .903 |
| Sex | 4.66 | 2.49 | .10 | 1.87 | .062 |
| Step 2: *F*(5, 323) = 6.75, *p* < .001, *R^2^* = .10 | | | | | |
| Fear of negative evaluation* | -9.41 | 2.64 | -.40 | -3.56 | < .001 |
| Social anxiety in general | 4.00 | 7.10 | .07 | 0.56 | .573 |
| Sex | 4.73 | 2.50 | .10 | 1.89 | .059 |
| Sex * Fear of negative evaluation | 3.61 | 3.29 | .13 | 1.10 | .273 |
| Sex * Social anxiety in general | -2.27 | 3.41 | -.08 | -0.67 | .505 |
| **2. Interpretation bias social picture task free evaluation difference** | | | | | |
| Step 1: *F*(3, 325) = 11.04, *p* < .001, *R^2^* = .09 | | | | | |
| Fear of negative evaluation* | -21.34 | 4.50 | -.33 | -4.74 | < .001 |
| Social anxiety in general | 7.84 | 11.10 | .05 | 0.71 | .481 |
| Sex | 10.55 | 6.76 | .08 | 1.56 | .119 |
| Step 2: *F*(5, 323) = 7.57, *p* < .001, *R^2^* = .11 | | | | | |
| Fear of negative evaluation* | -31.07 | 7.13 | -.48 | -4.36 | < .001 |
| Social anxiety in general | 8.09 | 19.17 | .05 | 0.42 | .673 |
| Sex | 11.41 | 6.75 | .09 | 1.69 | .092 |
| Sex * Fear of negative evaluation | 15.59 | 8.88 | .20 | 1.76 | .080 |
| Sex * Social anxiety in general | -1.52 | 9.21 | -.02 | -0.17 | .869 |
| **3. Interpretation bias textual vignette task difference** | | | | | |
| Step 1: *F*(2, 326) = 37.13, *p* < .001, *R^2^* = .19 | | | | | |
| Depressive mood* | -.35 | .13 | -.16 | -2.78 | .006 |
| Self-esteem* | .78 | .14 | .32 | 5.52 | < .001 |
| Step 2: *F*(5, 323) = 28.03, *p* < .001, *R^2^* = .30 | | | | | |
| Depressive mood | -.18 | .12 | -.08 | -1.49 | .138 |
| Self-esteem | .18 | .16 | .08 | 1.18 | .241 |
| Fear of negative evaluation* | -.41 | .10 | -.28 | -4.14 | < .001 |
| Social anxiety in general* | -.81 | .23 | -.22 | -3.45 | .001 |
| Sex | -.01 | .14 | -.00 | -0.04 | .969 |
| Step 3: *F*(7, 321) = 20.33, *p* < .001, *R^2^* = .31 | | | | | |
| Depressive mood | -.17 | .12 | -.08 | -1.40 | .164 |
| Self-esteem | .21 | .16 | .09 | 1.33 | .183 |
| Fear of negative evaluation | -.33 | .15 | -.23 | -2.21 | .028 |
| Social anxiety in general | -.57 | .39 | -.16 | -1.44 | .151 |
| Sex | -.02 | .14 | -.01 | -0.15 | .882 |
| Sex * Fear of negative evaluation | -.11 | .18 | -.06 | -0.63 | .531 |
| Sex * Social anxiety in general | -.12 | .19 | -.07 | -0.64 | .521 |
| *Note.* * Significant predictor in the model. | | | | | |

1. **Exploratory analyses: Positive versus negative bias**

**Introduction.** We also explored whether adolescents with social fears experience a negative interpretation bias because they over-interpret ambiguous situations in a negative way, or because they have a lack of interpreting situations in a positive way (Amir et al., 2012; Huppert et al., 2003; Steinman et al., 2020).

**Methods.** To investigate this, we calculated four extra interpretation bias indices. For the free evaluation part of the pictorial task, we calculated the mean score of the responses regarding the fit of the positive interpretations and the mean score of the responses regarding the fit of the negative interpretations. For the textual vignette task, we also calculated the mean score of the responses to the positive interpretations and the mean score of the responses to the negative interpretations.

**Results.** Four regression analyses were conducted, with the mean negative and mean positive scores of the free evaluation part of the social picture task and the textual vignette task as four dependent variables. Table 5 presents the regression coefficients of these analyses.

***Social Picture Task - Positive and Negative Interpretations.*** No covariates were taken into account for the regressions of the social picture task (due to similar reasons as described in the manuscript). Fear of negative evaluation and general social anxiety were entered as predictors in the first block (Bonferroni corrected α-levels for both regressions was .025). Results of these two regression analyses were comparable. Both models were significant. More fear of negative evaluation predicted the tendency to have less positive and more negative interpretations. General social anxiety symptoms were not related to positive or negative interpretations.

***Textual Vignette Task - Positive and Negative Interpretations.*** For the regression of the positive interpretations of the textual vignette task, self-esteem was added as a covariate in the first block. For the regression of the negative interpretations of the textual vignettes, both self-esteem and depressive mood were added as covariates in the first block. For both regression analyses, fear of negative evaluation and social anxiety in general were added as predictors in the second block. The Bonferroni corrected α-level for positive interpretation = .0167; and for negative interpretation = .0125.

For the positive interpretations, the model with the covariate only was significant. More self-esteem predicted more positive interpretations. After the social fear variables were added, self-esteem was still a significant predictor of positive interpretations and the model remained significant. Adding fear of negative evaluation and more general social anxiety to the model led to a significant change in model fit, *F*_change_ (2, 325) = 15.38, *p* < .001, *R^2^*_change_ = .07. Higher levels of fear of negative evaluation and more general social anxiety symptoms predicted lower positive interpretations with the textual vignette task.

For the negative interpretations, the model with the covariates only was significant. More depressive mood and lower self-esteem predicted more negative interpretations. After the main predictors were entered in the second block, the model remained significant. Adding the social fear variables to the model led to a significant change in fit, *F*_change_ (2, 324) = 19.33, *p* < .001, *R^2^*_change_ = .09. In this block, self-esteem was no longer a significant predictor of negative interpretations, but depressive mood was. Higher levels of fear of negative evaluation predicted more negative interpretations with the textual vignette task, but more general social anxiety was not a significant predictor.

**Discussion.** Individuals with higher levels of fear of negative evaluation are inclined to interpret situations in a negative way, but also lack positive interpretations of social situations on both the pictorial and textual vignette tasks. This finding was not replicated for more general social anxiety feelings. Specifically, social anxiety in general was not related to positive or negative interpretations measured with the pictorial task at all. For the textual task, individuals with more general social anxiety symptoms showed a lack of positive interpretations for situations, but did not have the tendency to interpret situations in a more negative way.

These findings are in line with researchers arguing that negative and positive interpretations are not opposite ends of the same continuum (Amir et al., 2012; Huppert et al., 2003; Steinman et al., 2020). Previous results were mixed, with some studies showing that adolescents with higher levels of social anxiety simultaneously rated positive interpretations as less likely and negative interpretation as more likely (Amir et al., 2012; Haller et al., 2016), while others found a stronger association between anxiety and the preference for threat interpretations (Huppert et al., 2003), or found that socially anxious individuals are mostly characterized by a lack of a preference for benign interpretations (Hirsch & Mathews, 2000). Our results further complicated the issue, as the link with positive versus negative interpretations was different for social anxiety in general and for the core fear of social anxiety, fear of negative evaluation, specifically. It is thus important to further investigate this issue by examining interpretation bias as a dimensional construct.

| **Table 6**  *Results of Four Multiple Regression Analyses Examining if Adolescents with Social Fears have a Lack of Positive Interpretations or Overuse Negative Interpretations (N = 329)* | | | | | |
| --- | --- | --- | --- | --- | --- |
|  | *B* | *B_SE_* | β | *t* | *p* |
| **1. Social picture task positive interpretations**  Step 1: *F*(2, 326) = 16.78, *p* < .001, *R^2^* = .09 | | | | | |
| Fear of negative evaluation* | -12.52 | 2.41 | -.36 | -5.19 | < .001 |
| Social anxiety in general | 8.45 | 5.94 | .10 | 1.42 | .156 |
| **2. Social picture task negative interpretations**  Step 1: *F*(2, 326) = 11.53, *p* < .001, *R^2^* = .07 | | | | | |
| Fear of negative evaluation* | 8.56 | 2.31 | .26 | 3.70 | < .001 |
| Social anxiety in general | -0.47 | 5.70 | -.01 | -0.83 | .934 |
| **3. Textual vignette task positive interpretations** | | | | | |
| Step 1: *F*(1, 327) = 58.50, *p* < .001, *R^2^* = .15 | | | | | |
| Self-esteem* | 0.52 | 0.07 | .39 | 7.65 | < .001 |
| Step 2: *F*(3, 325) = 31.47, *p* < .001, *R^2^* = .23 | | | | | |
| Self-esteem* | 0.23 | 0.09 | .17 | 2.66 | .008 |
| Fear of negative evaluation* | -0.15 | 0.06 | -.18 | -2.56 | .011 |
| Social anxiety in general* | -0.41 | 0.13 | -.21 | -3.09 | .002 |
| **4. Textual vignette task negative interpretations** | | | | | |
| Step 1: *F*(2, 326) = 25.16, *p* < .001, *R^2^* = .13 | | | | | |
| Depressive mood* | 0.32 | 0.08 | .24 | 3.98 | < .001 |
| Self-esteem* | -0.27 | 0.09 | -.18 | -2.95 | .003 |
| Step 2: *F*(4, 324) = 23.66, *p* < .001, *R^2^* = .23 |  |  |  |  |  |
| Depressive mood* | 0.24 | 0.08 | .17 | 2.97 | .003 |
| Self-esteem | 0.07 | 0.10 | .04 | 0.65 | .514 |
| Fear of negative evaluation* | 0.26 | 0.07 | .28 | 3.96 | < .001 |
| Social anxiety in general | 0.37 | 0.16 | .16 | 2.40 | .017 |
| *Note.* * Significant predictor in the model. | | | | | |
